# Structures and Metal-Binding Properties of *Helicobacter pylori* Neutrophil-Activating Protein with a Di-Nuclear Ferroxidase Center

**DOI:** 10.3390/biom4030600

**Published:** 2014-06-26

**Authors:** Hideshi Yokoyama, Satoshi Fujii

**Affiliations:** School of Pharmaceutical Sciences, University of Shizuoka, 52-1 Yada, Suruga-ku, Shizuoka 422-8526, Japan; E-Mail: satoshi_21@tenor.ocn.ne.jp

**Keywords:** *Helicobacter pylori*, neutrophil-activating protein, ferroxidase center, iron, zinc, cadmium, Dps, ferritin, solvent channel, pore

## Abstract

*Helicobacter pylori* causes severe diseases, such as chronic gastritis, peptic ulcers, and stomach cancers. *H. pylori* neutrophil-activating protein (HP-NAP) is an iron storage protein that forms a dodecameric shell, promotes the adhesion of neutrophils to endothelial cells, and induces the production of reactive oxygen radicals. HP-NAP belongs to the DNA-protecting proteins under starved conditions (Dps) family, which has significant structural similarities to the dodecameric ferritin family. The crystal structures of the apo form and metal-ion bound forms, such as iron, zinc, and cadmium, of HP-NAP have been determined. This review focused on the structures and metal-binding properties of HP-NAP. These metal ions bind at the di-nuclear ferroxidase center (FOC) by different coordinating patterns. In comparison with the apo structure, metal loading causes a series of conformational changes in conserved residues among HP-NAP and Dps proteins (Trp26, Asp52, and Glu56) at the FOC. HP-NAP forms a spherical dodecamer with 23 symmetry including two kinds of pores. Metal ions have been identified around one of the pores; therefore, the negatively-charged pore is suitable for the passage of metal ions.

## 1. Introduction

*Helicobacter pylori* is a Gram-negative bacterium that colonizes the human gastric mucosa and chronically infects up to 50% of the human population [1,2,3]. *H. pylori* infections cause severe diseases, such as chronic gastritis, peptic ulcers, and stomach cancers. The infiltration of neutrophils has been detected in the stomach mucosa of *H. pylori*-infected patients with chronic gastritis [4,5,6].

*H. pylori* neutrophil-activating protein (HP-NAP) is one of a number of virulence factors [7,8]. This protein has been shown to promote the adhesion of neutrophils to endothelial cells, and activates NADPH oxidase to produce reactive oxygen species (ROS) via a cascade of intracellular activation events [6,7,8,9,10]. HP-NAP binds to the outer membrane surface, which mediates binding to mucin or glycosphingolipids [7,10,11,12]. This protein can also stimulate the production of tissue factor and plasminogen activator inhibitor-2 by human monocytes [10,13]. HP-NAP can cross the endothelium to promote neutrophil adhesion *in vivo* [14] and can activate the underlying mast cells [7,15]. HP-NAP has also been shown to stimulate Th1 immune responses by inducing the production of cytokines, such as interleukin-12 (IL-12) and IL-23 [16,17,18]. HP-NAP is a major antigen in the immune response to *H. pylori* infections, and the majority of infected patients produce antibodies specific for HP-NAP [8,10,18]. Therefore, HP-NAP is a candidate for an anti-*H. pylori* vaccine [10,19,20], and is currently undergoing clinical trials [18,21].

Based on amino-acid sequence comparisons [22], HP-NAP belongs to the DNA-protecting proteins under starved conditions (Dps) family [23], which has significant structural similarities to the dodecameric ferritin family [24]. HP-NAP protects *H. pylori* from iron-mediated oxidative DNA damage by sequestering free iron [25,26], similar to Dps proteins, which protect DNA from oxidative damage [27]. Dps proteins are able to incorporate Fe^2+^ ions inside their dodecameric shell, and the incorporated Fe^2+^ ions are oxidized to Fe^3+^ ions at the ferroxidase center (FOC) located within the dodecamer. After that, the ions mineralize as hydrous ferric oxides (FeOOH) [28]. The presence of Fe^2+^ can lead to the generation of hydroxyl radicals through the Fenton reaction [28]. Dps proteins prevent the production of ROS such as hydroxyl radicals by sequestering Fe^2+^ ions, which protects DNA from oxidative damage by ROS [28]. Ferritin has been shown to detoxify and store iron ions by sequestering them, and binds to metals, such as Cd^2+^, Zn^2+^, Tb^3+^, or Ca^2+^, in addition to Fe^2+^ [29]. HP-NAP may bind metals other than iron given its similarity with dodecameric ferritin, however, to the best of our knowledge, this has not yet been examined.

HP-NAP is a dodecameric protein consisting of 17-kDa monomers, and has a spherical shell 9–10 nm in diameter with a hollow central core in which iron ions bind [22]. HP-NAP can bind up to 500 atoms of iron per dodecamer *in vitro* [22]. The crystal structure of HP-NAP from *H. pylori* strain 26695 (HP-NAP 26695) containing one iron ion per monomer was the first to be determined [30], and its structure was found to be similar to those of dodecameric Dps and dodecameric ferritins [24,31,32]. We recently determined the crystal structures of the apo form and metal-ion bound forms such as iron, zinc, and cadmium of HP-NAP from strain YS39 (HP-NAP YS39) [33,34]. This review focused on the structures of HP-NAP. We discussed the different metal-coordination patterns and structural rearrangements caused by metal-ion binding by comparing these structures. We also described the metal ion-uptake pathway.

## 2. Overall Structures of HP-NAP

### 2.1. Dodecameric Structure

HP-NAP has 144 amino-acid residues. The amino-acid sequences of HP-NAP from strains YS39 and 26695 were found to be almost similar [34]. HP-NAP YS39 differs from HP-NAP 26695 at four residues (E46G, V59A, I73L, and Y101H). His25, His37, Asp52, and Glu56 are perfectly conserved among HP-NAPs, dodecameric ferritin, and Dps proteins, and play important roles in metal-ion binding [34].

The monomer of HP-NAP is composed of a four-helix bundle (helices 1, 2, 3, and 4) with a fifth helix (helix 2–3) of seven residues (Leu69–Leu75) oriented almost perpendicular to the bundle. The secondary structure of HP-NAP was found to be similar to those of Dps proteins. The monomers of Fe-loaded HP-NAPs from strains YS39 and 26695 are almost identical, with the root-mean-square difference (r.m.s.d.) of the corresponding Cα atoms being 0.36 Å.

**Figure 1:**
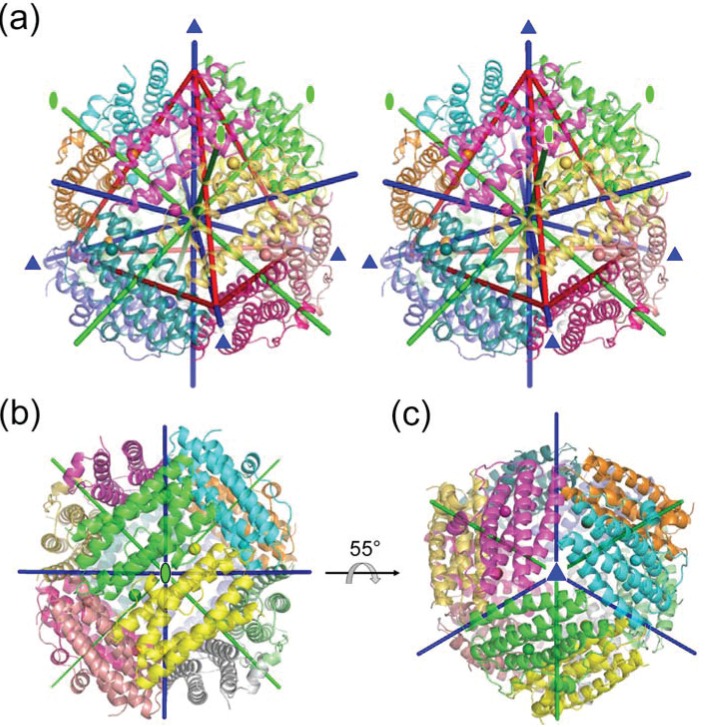
Overall structure of Fe-loaded HP-NAP YS39 (PDB ID: 3TA8). Each subunit with iron ions is shown in a different color. (**a**) Overall structure with a red tetrahedron to show the structure symmetry (stereoview). Green lines indicate two-fold axes, and blue lines indicate three-fold axes; (**b**) The view is from a two-fold axis; (**c**) The view is from one of three-fold axes.

A total of 12 protein subunits form a dodecamer, like a spherical shell, and this is approximately 90 Å in diameter (Figure 1). The internal cavity of the dodecamer is approximately 50 Å in diameter. The dodecamer adopts 23 symmetry. Hence, the four trimers are placed at the vertices of a tetrahedron, shown in red lines in Figure 1a. Three two-fold axes pass through the centers of the tetrahedron edge and the center of the structure. Four three-fold axes (Figure 1a,b) pass through the vertices of the tetrahedron and the centers of the opposite face. Therefore, there are two nonequivalent environments along the three-fold axes arranged as pores: One pore (pore I, described below) is present on the side of the vertices of the tetrahedron, and the other pore (pore II, described below) is present on the opposite side (Figure 1a). Figure 1c is viewed from pore I on the side of the vertices of the tetrahedron. This dodecameric structure was shown to be similar to Dps proteins [23,32] and *Listeria innocua* dodecameric ferritin [24]. The overall structure of HP-NAP YS39 is similar to those of other HP-NAPs and Dps proteins.

### 2.2. Crystal Packing

**Figure 2:**
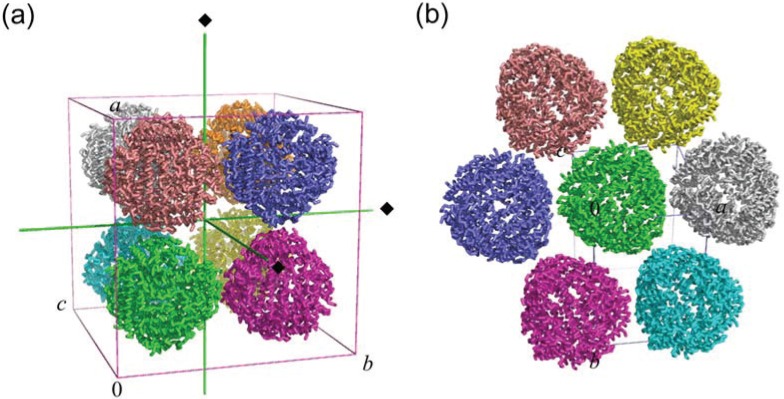
Crystal packing of Fe-loaded HP-NAPs from strains YS39 (PDB ID: 3TA8) (**a**) and 26695 (PDB ID: 1JI4) (**b**). Each dodecamer is shown in a different color. Green lines indicate four-fold axes. Each unit cell is also shown.

The space group of Fe-loaded HP-NAP from strain YS39 (*F*432) differs from that of strain 26695 (*P*2_1_). The Fe-loaded HP-NAP YS39 structure contains one monomer in the asymmetric unit, and the dodecameric structure is formed by a crystallographic symmetry relationship. One dodecamer makes crystal contacts with six neighboring dodecamers to give simple cubic packing with a crystallographic four-fold axis, as shown in Figure 2a. On the other hand, the Fe-loaded HP-NAP 26695 structure contains a dodecamer (12 monomers) in the asymmetric unit. One dodecamer makes crystal contacts with six neighboring dodecamers in the same plane and six additional neighboring dodecamers to give the characteristic pseudo-hexagonal sheet packing (Figure 2b) similar to that in the *Escherichia coli* Dps structure [23].

**Figure 3:**
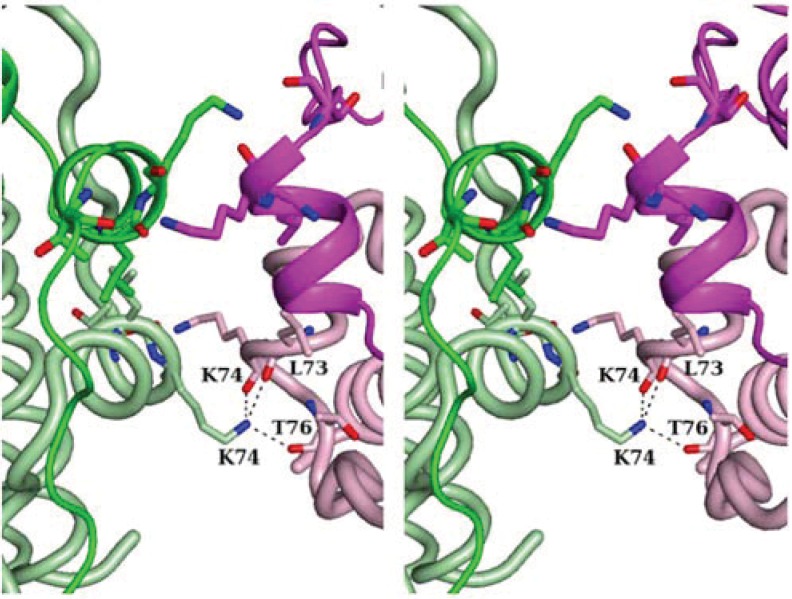
Stereo-representation of the molecular contacts between two neighboring dodecamers in Fe-loaded HP-NAP YS39. The Lys74 Nζ atom is hydrogen-bonded to the backbone O atoms of Leu73, Lys74, and Thr76 of the neighboring dodecamers.

**Figure 4:**
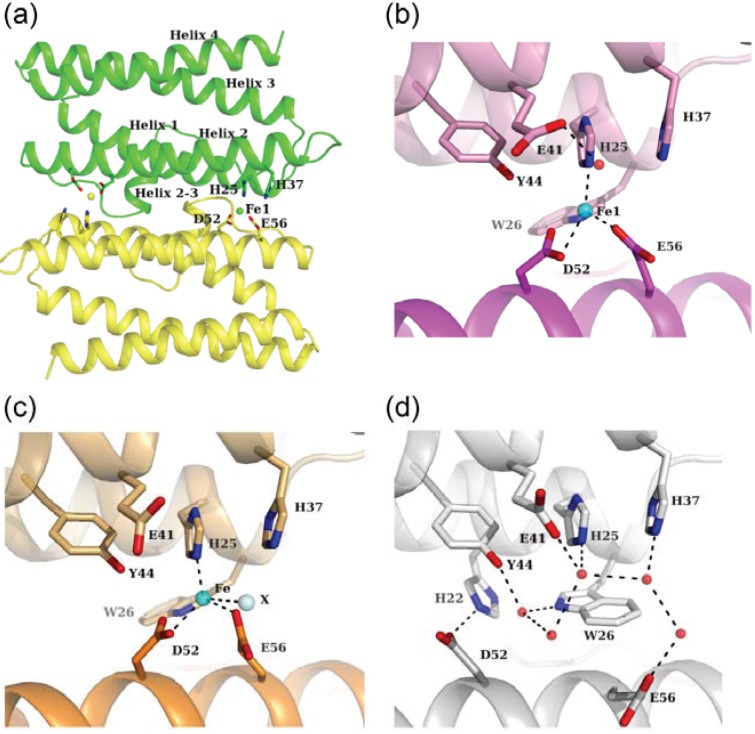
Ferroxidase center. (**a**) Two ferroxidase centers are located in the interface of the dimer in Fe-loaded HP-NAP YS39. His25 and His37 of one subunit (green) and Asp52 and Glu56 of the other subunit (yellow) are chelated to iron ions. Fe1 indicates the first iron ion. This figure is viewed from the inner side of the dodecamer; (**b**) A close-up view of the FOC in Fe-loaded HP-NAP YS39. Iron and water molecules are shown as cyan and red spheres, respectively. Dashed lines indicate coordinate bonds or hydrogen bonds; (**c**) A close-up view of the FOC in Fe-loaded HP-NAP 26695. An unidentified solvent molecule isshown as palecyan; (**d**) A close-up view of the FOC in the HP-NAP YS39 apo (PDB ID: 3T9J).

Inter-dodecamer contacts are made in Fe-loaded HP-NAP YS39 through the central regions of helices 2–3 of four subunits (Figure 3 and Figure 4a). There are four bridged stacks of Lys74 residues. Each Lys74 Nζ atom is hydrogen-bonded to the backbone carbonyl O atoms of Leu73, Lys74, and Thr76 of the neighboring dodecamers. The bridged stacks are stabilized by hydrophobic interactions with the neighboring alkyl side chains.

Due to different crystal packing between Fe-loaded HP-NAPs from strains YS39 and 26695, the solvent content of Fe-loaded HP-NAP YS39 is 65% (v/v) (and *V*_M_ value is 3.68 Å^3^/Da), whereas that of Fe-loaded HP-NAP 26695 is 55% (v/v) (and *V*_M_ value is 2.78 Å^3^/Da). A heavily solvated crystal lattice forming a wide water channel has been identified in Fe-loaded HP-NAP YS39, with the absence of masking contact with the adjacent dodecamers at the three-fold axis-related pore. The three-fold-related pore may encourage the entry of metal ions into the protein as described below. Crystal packing in Fe-loaded HP-NAP YS39 may be useful in metal ion soaking studies. Our group has previously determined the crystal structure of Zn-loaded HP-NAP YS39 using the soaking method [34].

Our group also determined the crystal structures of the apo form and iron-, zinc-, and cadmium-ion bound forms of HP-NAP YS39. All these crystals belong to the same space group, *F*432. Inter-dodecamer contacts have been identified through the bridged stacks of Lys74 in all the structures, and the distances between two Lys74 Cα atoms from the adjacent subunits were found to be almost identical: the distances between two Lys74 Cα atoms from adjacent subunits were 4.5 Å for the YS39 apo and 4.4 Å for the Zn-loaded YS39. However, the unit-cell dimension of the YS39 apo (*a* = 189.47 Å) is approximately 4 Å longer than that of Zn-loaded YS39 (*a* = 185.53 Å). Therefore, the dodecamer of the YS39 apo is longer in diameter than that of Zn-loaded YS39. The approximate volume of the internal cavity was calculated previously by the CASTp server [35]. The volume of the YS39 apo (8.8 × 10^4^ Å^3^) was found to be 25% larger than that of Zn-loaded YS39 (7.0 × 10^4^ Å^3^). The volume of *Salmonella* *enterica* Dps [32] is 5.3 × 10^4^ Å^3^, which is markedly smaller than that of Zn-loaded YS39; however, their subunits are similar to each other. These results indicate that the spherical shell-like dodecamers of HP-NAP and Dps proteins can easily change their sizes. Given ionic radii of metal ions, these volumes of the internal cavity of HP-NAP are sufficient to bind 500 atoms of iron per dodecamer as reported [22]. In the structure of the Fe-loaded YS39, however, only two iron ions per subunit were identified as described later [33].

## 3. Ferroxidase Center

### 3.1. Fe-Loaded and Apo Structures

Two ferroxidase centers (FOCs) are located between two subunits related by a two-fold axis (Figure 1b). In Fe-loaded HP-NAP YS39, an iron ion at one FOC is 21.7 Å away from the another iron ion at the other symmetry-related FOC (Figure 4a). This value is almost similar to that for other Dps proteins [36]. The inner side of the dodecameric shell consisting mainly of helices 2 and 4 has a strong negative charge, and is suitable for iron storage (Figure 4a). This is also a common feature of Dps proteins [30]. His25 and His37 are located on helices 1 and 2 of one subunit, respectively, and both Asp52 and Glu56 are located on helix 2 of the other subunit. These residues form a FOC, and an iron ion is located near these residues. The Fe-loaded HP-NAPs from strains YS39 and 26695 have similar FOC structures (Figure 4b,c). An unidentified solvent atom (perhaps Cl^−^ ion) is located near the iron ion in HP-NAP 26695, whereas there is no corresponding atom in HP-NAP YS39. In both structures, the iron ions are coordinated in a distorted tetrahedral manner to the side chains of His25, Asp52, Glu56, and the solvent atom that is absent in HP-NAP YS39. FOCs commonly contain di-iron sites, with the two ions being separated by a distance of approximately 3 Å. However, no second iron ion was observed in the FOCs of Fe-loaded HP-NAPs as discussed below.

Figure 4d shows the FOC in the HP-NAP YS39 apo. Several water molecules form a hydrogen bonding network. Based on the comparison between Fe-loaded and apo structures (Figure 4b,d), the conformations of Trp26, Asp52, and Glu56 are largely changed. The conformations of His25, His37, Glu41, and Tyr44 are very similar to each other in the two structures, although the direction of the carboxylate of Glu41 is changed. The superposition of the equivalent Cα atoms of the dimeric α-helical bundles between the Fe-loaded and apo structures yields a relatively large r.m.s.d. of 0.92 Å. The r.m.s.d.s of helices 1, 2, 3, and 4 are 0.61, 1.29, 0.90, and 0.65 Å, respectively, which indicates that the displacement of helix 2 is significantly larger than those of other helices. Iron binding by two residues, Asp52 and Glu56, which are located on helix 2, may promote the displacement of helix 2. The binding of the iron ion to the FOC in HP-NAP YS39 apo leads to the following conformational changes. The Trp26 side chain switches by approximately 180° along with the Cβ−Cγ bond (χ_2_ angle). It rotates the indole ring, and then moves away from the FOC. The carboxyl side chains of Asp52 and Glu56 move toward the resultant vacant space in the FOC, and can be chelated by the iron ion. In the apo structure, the side chain of Asp52 is stabilized by the formation of a salt bridge to His22, and the side chain of Glu56 is also stabilized by hydrogen-bonding to a water molecule. Trp26, in addition to His25, His37, Asp52, and Glu56, is well conserved among HP-NAPs and Dps proteins, and may play an important role in the conformational changes that occur upon binding of iron ions [33,34].

### 3.2. Zn-Loaded and Cd-Loaded Structures

**Figure 5:**
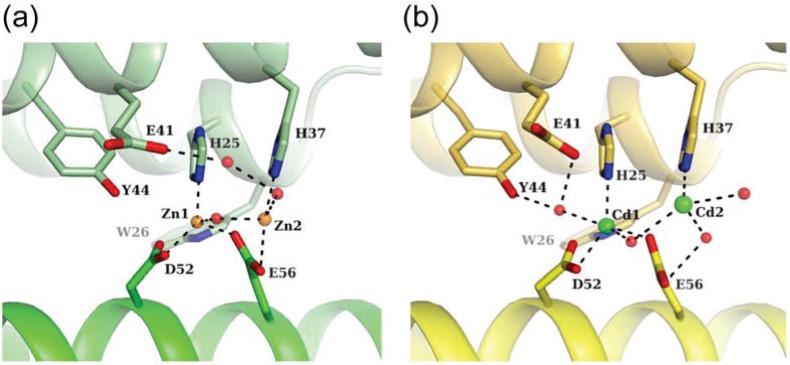
Close-up views of the ferroxidase center. (**a**) Zn-loaded HP-NAP YS39 (PDB ID: 4EVB). Zinc and water molecules are shown as orange and red spheres, respectively. Dashed lines indicate coordinate bonds or hydrogen bonds; (**b**) Cd-loaded HP-NAP YS39 (PDB ID: 4EVC). Cadmium and water molecules are shown as green and red spheres, respectively.

Two metal ions are observed in the FOC in the Zn-loaded and Cd-loaded HP-NAP YS39 (Figure 5). The first zinc ion, Zn1 is coordinated in a tetrahedral manner to the side chains of His25, Asp52, Glu56, and a water molecule that bridges two zinc ions (Figure 5a). The second zinc ion, Zn2 is coordinated in a distorted tetrahedral manner to the side chains of His37, Glu56, and two water molecules. On the other hand, the first cadmium ion, Cd1 is coordinated in a trigonal-bipyramidal manner, in which the triangle is formed by the side chains of His25, Asp52, and a water molecule bridging two cadmium ions, and the corner of the pyramid is formed by the side chain of Glu56 and another water molecule (Figure 5b). The second cadmium ion, Cd2 is coordinated in a distorted octahedral manner to the side chain of His37 and three water molecules, with two sites remaining unoccupied. Zinc ions are more likely to coordinate in a tetrahedral arrangement. Cadmium ions are also more likely to coordinate in both tetrahedral and octahedral arrangements, but also exhibit a versatile coordination geometry [37]. Cadmium ions were found to be coordinated in a trigonal-bipyramidal manner in the structures of phosphotriesterase [38], phosphoglucomutase [39], and thermolysin [40].

### 3.3. Comparison of Metal Coordination

The first metal binding sites (Fe1, Zn1, and Cd1) are very similar to each other in the two structures among the Fe-loaded, Zn-loaded, and Cd-loaded structures (Figure 4b,c and Figure 5a,b). However, the second metal binding sites are different. No second iron has been reported in the FOC of Fe-loaded HP-NAP YS39. An unidentified solvent atom is located near the second metal binding site in Fe-loaded HP-NAP 26695, and the Fe−X distance is 3.1 Å. The Zn1−Zn2 distance has shown to be 3.3 Å, which is similar to the di-zinc distance in the FOC of the Dps proteins, *Streptococcus pyogenes* Dpr (3.5 Å) [41] and *S. suis* Dpr (3.3 Å) [42]. The Cd1–Cd2 distance is 4.2 Å, which is longer than the di-zinc distance. The mean first metal-ligand bond distances of HP-NAP YS39 structures were reported to be 2.33 Å for Fe1, 2.13 Å for Zn1, and 2.38 Å for Cd1 [34]. The mean metal-ligand bond distances in metalloproteins in the case of His, Asp, and Glu were 2.14 Å for Zn^2+^ and 2.39 Å for Cd^2+^ [37], which were comparable to the values of HP-NAP YS39. The longer metal-ligand bond distances for Cd1 than Zn1 may result in a Cd1−Cd2 distance that is longer than the Zn1–Zn2 distance. The mean second metal-ligand bond distances of HP-NAP YS39 structures were shown to be 2.41 Å for Zn2 and 2.62 Å for Cd2 [34], both of which are longer than the first metal-ligand bond distances. Furthermore, the *B*-factor for Zn1 is 48.8 Å^2^, which is lower than that for Zn2, 51.2 Å^2^. The *B*-factor for Cd1 is 20.6 Å^2^, which is lower than that for Cd2, 42.1 Å^2^. These results indicate that the second metal coordination is weaker than the first one. Zinc ions have been used as an oxidation stable replacement for iron ions [41]. Di-zinc binding to FOC in HP-NAP YS39 indicates that di-iron FOC can be formed. To obtain crystals of Fe-loaded HP-NAP YS39, 10 mM iron (II) sulfate was used. However, Fe^2+^ ions are thought to be oxidized to Fe^3+^, due to long exposure to oxygen during crystallization [33,34]. As described in Dps proteins [28,36,41], di-iron sites are only occupied by Fe^2+^, which is rapidly oxidized to Fe^3+^. The second iron ion is loosely coordinated, and released from the FOC just after Fe^2+^ is oxidized to Fe^3+^. Based on these findings, there appears to be no second iron in the FOCs of Fe-loaded HP-NAPs from strains YS39 and 26695 (Figure 4b,c).

## 4. Metal Ion Entry through the Three-Fold Pore

### 4.1. Pore I

**Figure 6:**
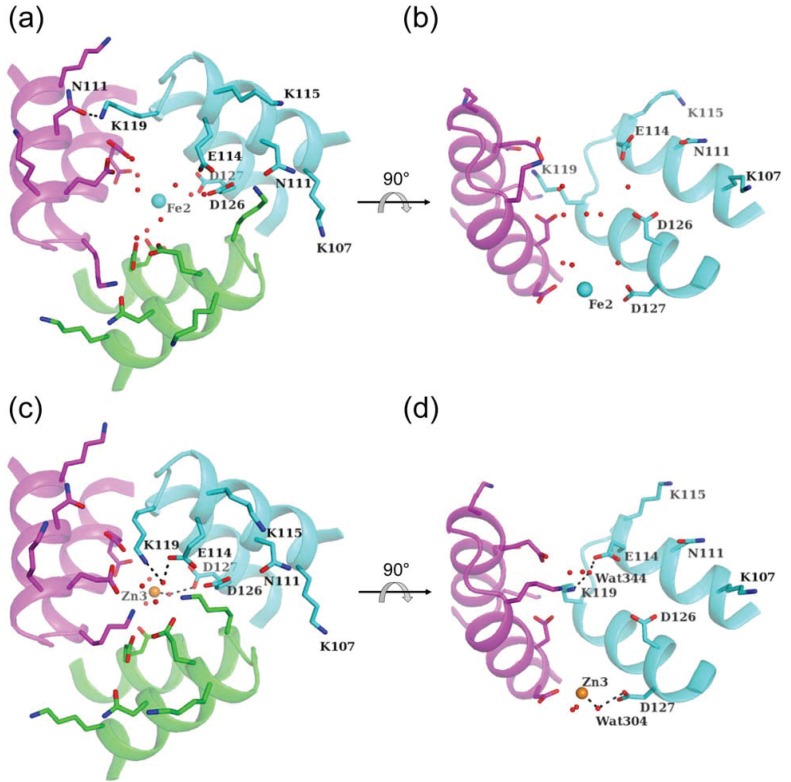
Pore I along the three-fold axis. Three-fold-axis-related trimers (cyan, magenta, and green) in Fe-loaded HP-NAP YS39 (**a**, **b**) and Zn-loaded HP-NAP YS39 (**c**, **d**). Iron, zinc, and water molecules are shown as cyan, orange, and red spheres, respectively. The key residues forming pore I are shown in sticks. Dashed lines indicate coordinate bonds or hydrogen bonds. Fe2 indicates the second iron ion of Fe-loaded HP-NAP YS39. Zn3 indicates the third zinc ion of Zn-loaded HP-NAP YS39. The view is from outside of the spherical shell (**a**, **c**) and is from the side (**b**, **d**). One polypeptide chain (green) is omitted for clarity in (**b**, **d**).

The HP-NAP dodecamer contains two types of three-fold axis-related pores. One pore (pore I) is strongly hydrophilic and has a negatively charged environment that may guide metal ions toward the internal cavity (Figure 1c and Figure 6). Three layers of negatively charged residues, Glu114, Asp126, and Asp127, are located along with the pore from the outside to the inside (Figure 6b,d). These residues are conserved among HP-NAPs. Such a funnel-like pore along with the three-fold axis is filled with a network of solvent molecules, and this is suitable for metal ion entry by electrostatic interactions. This type of pore has been reported in Dps proteins [32,43,44,45,46] and *L. innocua* dodecameric ferritin [24].

Fe and Zn atoms are located inside the pore in Fe-loaded and Zn-loaded HP-NAP YS39 structures, and the location of the two ions are almost the same. No ordered cadmium ion has been observed in Cd-loaded HP-NAP YS39. The Zn atom is coordinated to three symmetrically-related water molecules (Wat304), each of which is hydrogen-bonded to the side chain of Asp127. Although no water molecules are observed around Fe2, the Fe atom may be chelated to disordered water molecules that connect to the side chain of Asp127 by hydrogen-bond networks. Such a flexible rearrangement may be suitable for trapping and transferring metal ions into the internal FOC.

The positively-charged residues, Lys107, Lys115, and Lys119, around the negatively-charged pore are positioned outside the entrance to the pore, and may function as an effective electrostatic guide for metal-ion transfer. The side chain of Lys119 is hydrogen-bonded to the side chain of Asn111 of the adjacent subunit in Fe-loaded HP-NAP YS39 (Figure 6a), which is the same in the Cd-loaded one. However, the side chain of Lys119 is hydrogen-bonded to a water molecule (Wat344) in the Zn-loaded HP-NAP YS39, and this is hydrogen-bonded to the side chain of Glu114 (Figure 6c). In Zn-loaded HP-NAP YS39, the distance between Wat344 and Wat304 is 12.6 Å, which corresponds to the length of the pore. The intersubunit distances between two corresponding atoms of the three subunits are as follows; Glu114 Oε2 (5.4 Å), Lys119 Nζ (4.5 Å), Asp126 Oδ2 (8.1 Å), and Asp127 Oδ1 (6.9 Å). The flexible side-chain of Lys119, which forms the narrow pore, may function as a filter bulb to help to drive metal ions inside the pore [34].

### 4.2. Pore II

The other pore (pore II) is located just opposite of pore I, as described in Section 2.1, and is composed of Thr31, Asp32, and Asn35. According to the intersubunit distances between two corresponding atoms of the three subunits, the narrowest distance is Asn35 Nδ2 (3.0 Å) in Fe-loaded HP-NAP YS39, and Asn35 Nδ2 (3.1 Å) in Zn-loaded HP-NAP YS39. Therefore, the size of pore II is smaller than that of pore I. No metal ions were observed in pore II in the HP-NAP structures determined previously.

By comparing the average *B*-factors of main-chain atoms, the regions around pore I have higher *B*-factors than those of the other regions in Fe-loaded, Zn-loaded, and Cd-loaded structures. The regions around pore I in the apo structure have been shown to have lower *B*-factors than the other regions [34]. According to these findings, ions and water molecules may frequently enter into and exit from pore I, which is suitable for the passage of metal ions.

## 5. Other Metal-Binding Sites

This review focused on metal bindings to the ferroxidase center and the three-fold axis related pore. However, other metal-binding sites have also been observed inside the dodecamer.

In the Fe-loaded HP-NAP YS39, two iron ions per subunit are identified in the FOC and inside the pore I as described [33]. In the Zn-loaded HP-NAP YS39, four zinc ions per subunit are identified: two zinc ions are in the FOC, a zinc ion is inside the pore I, and another one zinc ion is inside the protein shell, coordinating to Glu41 and Glu45. In the Cd-loaded HP-NAP YS39, four cadmium ions per subunit are identified: two cadmium ions are in the FOC, and another two cadmium ions are inside the protein shell. One cadmium ion is located at almost the same site as the zinc ion, and is coordinated to Glu42 and Glu45. Additionally, the other cadmium ion is coordinated to Asp49 and Asp53 [34]. These metal-binding sites other than the FOC and pore I are also involved in the metal-binding function.

## 6. Neutrophil Activation

**Figure 7:**
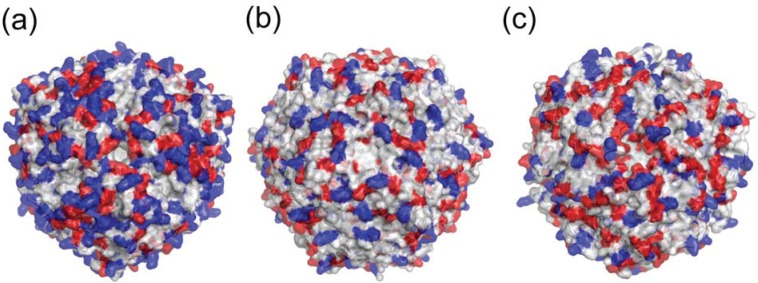
Molecular surface respresentations of HP-NAP and Dps proteins. Negatively charged residues (Asp and Glu) are colored red and positively charged residues (Lys, Arg, and His) blue. (**a**) The Fe-loaded HP-NAP YS39 dodecamer; (**b**) *E. coli* Dps dodecamer (PDB ID: 1DPS); (**c**) *L. innocua* ferritin dodecamer (PDB ID: 1QGH).

HP-NAP is capable of activating human neutrophils [8,9]. Major differences were found when electrostatic surface potentials were compared between HP-NAP and Dps proteins. The number of charged residues was found to be fewer in *E. coli* Dps [23] than in HP-NAP (Figure 7a,b). The number of positively charged residues was higher in HP-NAP than in *L. innocua* dodecameric ferritin [24] (Figure 7a,c). The HP-NAP surface is characterized by the large presence of positively charged residues, which are not shared by the other Dps protein members (Figure 7a) [30]. The positively-charged residues of several proteins, including some chemokines, appear to play a role in the activation of neutrophils [30,47,48]. Thus, a large number of positively-charged residues on the surface of HP-NAP may be responsible for its neutrophil-activating properties [30]. The C-terminal region containing helix 2–3 (residues 69–75) and helix 3 (residues 89–114) of HP-NAP may be critical in stimulating neutrophil activation [49]. Helix 2–3 and helix 3 are exposed to the outer surface of the dodecamer. Additional mutational studies with site-directed mutagenesis are required to identify the amino acids involved in stimulating neutrophil activation [49].

NapA from *Borrelia burgdorferi* is a Dps-like protein with specific immunomodularoty properties via Toll-like receptor (TLR) 2 [50]. Both NapA from *B. burgdorferi* and HP-NAP from *H. pylori* have been demonstrated to be TLR2 agonists [16,51]. TLR2 plays a crucial role in innate immunity by recognizing several kinds of different microbial molecules [52]. The external surface of the extracellular region of TLR2 possesses both positively and negatively charged residues spread all around the surface. NapA and HP-NAP also possess exposed surfaces with positive and negative charges. It is hypothesized that NapA and HP-NAP directly interact with the extracellular region of TLR2 [50].

## 7. Conclusions

*H. pylori* neutrophil-activating protein (HP-NAP) is an iron storage protein that promotes the adhesion of neutrophils to endothelial cells [8,9,22]. The crystal structures of the apo form and metal-ion bound forms such as iron, zinc, and cadmium of HP-NAP have been determined [30,33,34]. A total of 12 protein subunits, composed of four-helix bundles, form a spherical shell-like dodecamer with 23 symmetry. These structures are similar to those of Dps proteins. The inner side of the dodecameric shell has a strong negative charge, and is suitable for iron storage. Metal ions bind at the di-nuclear ferroxidase center by different coordinating patterns. In comparison with the apo structure, metal loading causes a series of conformational changes in conserved residues among HP-NAP and Dps proteins (Trp26, Asp52, and Glu56) at the FOC [33]. The Zn-loaded and Cd-loaded HP-NAP structures have revealed the di-nuclear binding mode in which the second ion is more weakly coordinated than the first at the FOC, whereas the Fe-loaded HP-NAP structure revealed the mono-nuclear mode [34]. The second iron ion is loosely coordinated, and may be released from the FOC just after Fe^2+^ is oxidized to Fe^3+^. Zinc and cadmium ions bind to the FOC, which indicates that HP-NAP can store zinc and cadmium ions in addition to iron ions. Metal ions are found around one of the pores; therefore, the negatively-charged three-fold-related pore is suitable for the passage of metal ions.
